# Tele-SPACE: group parent-based treatment for pediatric anxiety via telemedicine in a public health clinic

**DOI:** 10.3389/fpsyt.2025.1570497

**Published:** 2025-08-19

**Authors:** Naama de la Fontaine, Tal Cohen, Nimrod Hertz-Palmor, Shirel Dorman-Ilan, Noa Rubin, Gal Itav, Shlomit Tsafrir, Yael Carmel, Hana Weisman, Doron Gothelf, Eli R. Lebowitz

**Affiliations:** ^1^ The Edmond and Lily Safra Children’s Hospital, Sheba Medical Center, Ramat Gan, Israel; ^2^ Child Study Center, Yale School of Medicine, New Haven, CT, United States; ^3^ School of Psychology, The Gershon Gordon Faculty of Social Sciences, Tel Aviv University, Tel Aviv, Israel; ^4^ Medical Research Council (MRC) Cognition and Brain Sciences Unit, University of Cambridge, Cambridge, United Kingdom; ^5^ Baruch Ivcher School of Psychology, Reichman University, Herzliya, Israel; ^6^ The Faculty of Medical and Health Sciences, Tel-Aviv University, Tel Aviv, Israel; ^7^ Sagol School of Neuroscience, Tel Aviv University, Tel Aviv, Israel

**Keywords:** pediatric anxiety disorders, parent guidance, telemedicine, parent group treatment, remote treatment, public health

## Abstract

**Introduction:**

Supportive Parenting for Anxious Childhood Emotions (SPACE) is an evidence-based treatment for parents of children with anxiety disorders and/or obsessive-compulsive disorder (OCD). Given the many barriers to accessing such evidence-based treatments, we evaluated for the first time the application of group-based SPACE, delivered to parents via telemedicine within a public health outpatient setting.

**Methods:**

In this single arm retrospective analysis of routine-care data participants, recruited from a hospital-based anxiety clinic, were mothers (N=50) of fifty children, ages 6.7-18.0 years (mean 11.2 ± 3.1), diagnosed with an anxiety disorder and/or OCD. Parent and child self-report measures assessed the impact of treatment on child anxiety symptoms, parental accommodation, parental anxiety and depression. Treatment feasibility, acceptability and satisfaction were assessed.

**Results:**

Post treatment, significant reductions were evident in child self-reported separation anxiety symptoms (p =0.008), mother-reported child anxiety symptoms (p=0.002), maternal accommodation (p=0.006), anxiety (p=0.004) and depressive symptoms (p=0.011). Treatment proved feasible, with completion rates of 77.3%, and participants reported high levels of satisfaction with the telemedicine format.

**Discussion:**

This is the first study of group-based SPACE over telemedicine. Results support the utility of this modality for overcoming treatment barriers in public health settings with highly heterogenous populations.

## Introduction

Pediatric anxiety disorders are highly prevalent, affecting up to 30% of children (1–3), and frequently co-occur with other mental health conditions such as mood disorders, ADHD, learning disorders, OCD, and substance disorders (4, 5). This high comorbidity contributes to caregiver strain and increased demand for public health services, highlighting the need for accessible, evidence-based treatments (6).

Cognitive Behavioral Therapy (CBT) is the standard treatment for pediatric anxiety disorders, but often faces considerable access and engagement barriers, including child treatment refusal and attrition (7–14). Given the critical role parents play in their child’s anxiety (15, 16) parent-based treatments have emerged as efficacious alternatives, showing positive treatment outcomes along with comparable retention rates (17–19) Telemedicine treatment has been linked to significantly fewer parent-perceived barriers to treatment (20), reducing travel time, eliminating geographic barriers, and offering greater scheduling flexibility (21, 22). Taken together, this highlights the value of parent-based interventions delivered via telemedicine.

Supportive Parenting for Anxious Childhood Emotions (SPACE) is a parent-based treatment informed by the important role parents’ perceptions and reactions play in maintaining child anxiety (23). SPACE aims to reduce parental accommodations, behaviors intended to prevent or minimize child distress, which can inadvertently maintain or exacerbate anxiety over time (24). Parental accommodation is also associated with increased maternal distress, which has been linked to child anxiety (25–27). Randomized controlled trials have demonstrated that SPACE is efficacious (28) and comparable in efficacy to child-based CBT (29). Our team adapted SPACE to a group format, delivered in person, and found significant reductions on several parent-reported measures of child anxiety and OCD symptom severity, accommodation, family power struggles, and parent helplessness (30).

The COVID-19 pandemic led to significant rise in pediatric anxiety, straining psychiatric services (30, 31), and accelerating the shift from in-person treatment to more accessible telemedicine options (5). Telemedicine has demonstrated effectiveness in various mental health conditions and populations (32–34), and significantly enhances access to care (35, 36). Video teleconference *group* treatments have likewise been found to be effective and feasible alternatives to in-person treatment (37), warranting further evaluation to optimize telehealth applications (38). Despite advances in telemental health, to our knowledge, group-based SPACE has not yet been adapted to telemedicine. This may be attributable to a general trend of returning to in-person treatment delivery following the COVID-19 pandemic, (39) as well as documented challenges in maintaining therapist motivation and engagement for telemedicine-based interventions, particularly in group formats (40). Nevertheless, the persistent rise in pediatric anxiety disorders post-pandemic (41) and the resulting strain on public health systems highlight the ongoing need for scalable, accessible evidence-based treatments, including group-based modalities, within telehealth frameworks (42). These factors, together with promising outcomes from in-person group-based SPACE interventions, provided the impetus for the current study.

### The current study

This single-arm, retrospective analysis explores a novel implementation of group SPACE treatment via telemedicine in a heterogeneous sample within a public health setting, assessing its feasibility and effectiveness. As part of the routine care provided by the Safra Children’s Hospital Anxiety Clinic, group-based SPACE treatment was offered to all parents. Fifty mothers of 50 children and adolescents (ages 6.7–18 years) diagnosed with anxiety disorders and/or OCD participated in SPACE, which was delivered remotely in group settings. This retrospective analysis was conducted on routine care data, as participants were not enrolled in a clinical trial. The aims of the analysis were to assess a) the acceptability, feasibility, and satisfaction of telemedicine-delivered SPACE groups; b) preliminarily, its clinical efficacy in reducing children’s anxiety symptoms; and c) its impact on parental stress and accommodation. We hypothesized high feasibility, acceptability and satisfaction, reductions in child anxiety, parental accommodation, and parental symptomatology.

## Methods

The Sheba Medical Center Institutional Review Board (SMC-0635-23) approved the study and waived informed consent for the retrospective analysis of anonymous data that was routinely collected for clinical purposes at the outpatient Anxiety Clinic.

### Participants

This uncontrolled, single-arm, retrospective study included 50 mothers of 50 children (ages 6.7–18 years) diagnosed with an anxiety disorder and/or OCD who participated in remotely delivered group SPACE treatment. Treatment was offered to all parents. Nevertheless, the current study included mothers only, as the majority of participating fathers (N=27), despite mostly consistent engagement, did not complete pre- and post-treatment questionnaires (22.22% completion rate). Demographic data, including marital status, employment and education level for all recruited parents are presented in **Table 1**. Children’s mean age was 11.2 (range 6.7-18, SD = 3.1), and 26 (52.0%) were assigned female sex at birth (see **Table 1** for demographic details). The most common child anxiety disorder was generalized anxiety disorder (70.0%), yet comorbidity was common as is typical, and 68.0% of children were diagnosed with more than one disorder (comorbidity rates ranged from 1 to 3.) Non-anxiety comorbidity was particularly high for ADHD (44.0%) (see **Table 2**).

**Table 1:** Patient demographic data (N=50).

Characteristic	*n*	%
Age, years (M ± SD)	(11.2 ± 3.1)	
Gender		
Females at birth	26	52.0
Males at birth	24	48.0
Parents’ marital status		
Single	1	2.0
Married/partnered	41	82.0
Divorced	7	14.0
Widow/er	1	2.0
Parents’ employment status		
Both parents employed	45	90.0
Only 1 parent employed	4	8.0
Both parents unemployed	1	2.0
Parents’ educational level		
Doctoral degree	1	2.0
Master’s degree	9	18.0
Bachelor’s degree	19	38.0
Professional/Technical studies	8	16.0
High school or lower	13	26.0

**Table 2:** Patient diagnoses at baseline (N=50).

Diagnoses	*n*	%
Anxiety/OCD diagnoses		
Anxiety diagnoses*	46	92.0
GAD	35	70.0
Separation anxiety	8	16.0
Social anxiety	8	16.0
Panic disorder	1	2.0
Specific phobia	2	4.0
Selective mutism	2	4.0
OCD	4	8.0
OCD & other anxiety disorder	6	12.0
Additional comorbidities		
ADHD	22	44.0
Learning disorders	4	8.0
Conduct/ODD	3	6.0
Depression	1	2.0
Eating disorders	1	2.0
Speech/Language disorders	2	4.0
Adjustment disorder	1	2.0
Comorbidity	34	68.0

Average diagnoses were 2.1 ± 0.9 (*Range =* 1-4). *Percentages exceed 100% as many children carried multiple diagnosis. GAD, generalized anxiety disorder; ADHD, attention-deficit/hyperactivity disorder; ODD, oppositional defiant disorder.

Inclusion criteria for participating in group SPACE treatment were: a) child receiving a primary DSM-5 (45) diagnosis of an anxiety disorder or OCD; b) child residing with participating parent at least 50% of the time; and c) at least one parent committed to consistent attendance. Exclusion criteria for participating in group SPACE treatment were: a) child diagnosed with a psychotic disorder, autism spectrum disorder, or intellectual disabilities; b) parental or familial dysfunction that could hinder effective engagement (e.g., parental intellectual disabilities, substance abuse, psychotic disorder; unstable living or custody arrangement); c) high risk of self-harm or harm to others by the child; and d) concurrent psychosocial child-or parent-based anxiety treatment. There were no age-based inclusion or exclusion criteria. All eligible families were offered treatment regardless of child age, in line with common practices in public health settings where heterogeneous group composition helps reduce wait times and reflects real-world service demands (43, 44).

### Procedure

Participants underwent an in-person diagnostic interview conducted by a senior psychiatrist or psychologist. During the interview, those diagnosed with an anxiety disorder and/or OCD according to DSM-5 criteria (45) received psychoeducation on child anxiety disorders, and the SPACE treatment protocol (23) and were subsequently referred to the Child Anxiety Disorders and OCD Clinic. After agreeing to participate in SPACE treatment, parent and child self-report baseline measures were sent via separate secure links (Time 1).

The intervention consisted of 16 weekly sessions, each approximately 60 minutes in length, delivered remotely via video conference. Sessions were conducted in a group format (6–10 parent pairs or individuals) and followed the structured SPACE protocol developed by Lebowitz et al. (23), which targets reductions in parental accommodation to child anxiety. Each session focused on building parental awareness of accommodation patterns, providing psychoeducation about anxiety, enhancing parent self-regulation, and implementing specific behavioral changes to reduce accommodation. Treatment fidelity was monitored using a structured session checklist completed by group facilitators after each session. Weekly supervision was provided to ensure adherence to protocol, during which fidelity checklists were reviewed. In addition to facilitator-completed fidelity checklists, parent adherence to the SPACE protocol was supported through multiple structured strategies. Each week, parents completed standardized monitoring worksheets detailing accommodation behaviors, implementation of behavioral changes, and progress on tasks such as crafting supportive statements and announcement letters. Worksheets were submitted to group clinicians, who reviewed them prior to each session and provided individualized feedback. Parents often revised their letters or scripts based on this input, and shared them aloud in group sessions for further peer and clinician feedback. Role-play and simulation exercises were also used during sessions to model and reinforce key intervention strategies, including non-accommodative responses and effective delivery of messages to the child. These components allowed for ongoing monitoring, iterative skill refinement, and enhanced fidelity to the SPACE model, consistent with the framework outlined by Lebowitz et al. (23).

Participants who struggled with group pace, as evidenced by more than one missed session or noncompliance with homework assignments (i.e., worksheets) were offered individual “booster” sessions for additional support. Booster sessions did not introduce new material but focused on reviewing and clarifying concepts covered in the group sessions, addressing gaps or working through individual challenges. The majority of participants (74%) did not require booster sessions. Of the 26% who did, most received a single session, while only 3 participants (6%) required two sessions.

At treatment completion, parents and children received secure links to post-treatment questionnaires (Time 2). Feasibility was evaluated based on parent participation rates, attendance, treatment adherence, and completion. Acceptability was assessed via a parents’ response to a questionnaire assessing their satisfaction with the telemedicine platform was assessed through.

### Measures

All measures used in the study have been well-validated in Hebrew, ensuring their reliability and appropriateness for the target population.

#### Parental accommodation

Parental accommodation was assessed using the child- and parent-rated Family Accommodation Scale-Anxiety (FASA). The FASA consists of 13 items evaluating parents’ participation in symptom-driven behaviors (items 1–5), modifications to family routines (items 6–9), parent distress related to the accommodations (item 10), and short-term child consequences of non-accommodation (items 11–13). All items are scored on a 5-point Likert scale (0 = ‘not at all’ to 4 = ‘everyday’), with the first nine items summed to calculate the total accommodation score. Higher scores indicate greater parental accommodation (46).

#### Parental anxiety and depression symptoms

Parents completed the: 1) Beck Anxiety Inventory (BAI), a 21-item self-report measure for assessing anxiety severity in adults (47); and 2) Beck Depression Inventory (BDI), a 21-item self-report questionnaire for determining the presence and severity of depressive symptoms in adults (48).

#### Parent treatment satisfaction

Satisfaction with treatment was assessed using the Telemedicine Satisfaction Questionnaire (TSQ) (49)**-** a 14-item self-report questionnaire rated on a 5-point Likert scale (0-4) commonly used to assess treatment usefulness, satisfaction, and quality of interaction between patient and clinician over telemedicine (50).

#### Children’s anxiety symptoms

Children’s anxiety symptoms were assessed using the Screen for Childhood Anxiety Related Emotional Disorders (SCARED), a tool commonly used to assess anxiety symptoms through both parent and child self-reports. The SCARED consists of 41 items rated on a 3-point Likert scale (0-2) and includes five subscales: generalized anxiety, social anxiety, panic/somatic, separation anxiety, and school avoidance. A total score of 25 or above indicates clinically significant anxiety (51, 52).

#### Children’s OCD symptoms

Children’s OCD symptoms were assessed using the Obsessive-Compulsive Inventory – Revised (OCI) (53), a 18 items self-report questionnaire, with each item scored on a 5-point Likert scale. The OCI measures OCD symptoms across six subscales including washing, hoarding, obsessing, ordering, checking, and neutralizing. The total score for the OCI ranges from 0 – 60, with higher scores indicative of more severe OCD symptoms. A cutoff score of 12 indicates the likelihood of an OCD diagnosis. The OCI has demonstrated adequate test-retest reliability (53, 54).

#### Sociodemographic data

Sociodemographic data and patient diagnoses were collected through a diagnostic interview conducted before treatment initiation. The interview gathered the following information: Marital status, Employment status and Educational level. This interview also included an assessment of the patient’s mental health history and current symptoms to establish or confirm diagnoses according to DSM-5 criteria.

### Data analysis

Descriptive statistics were used to examine treatment acceptability and feasibility, encompassing dropout rates, session attendance, and satisfaction with telemedicine. One-tailed paired-sample t-tests were employed to assess pre-to-post treatment symptom reduction across various measures^1^, including child’s anxiety symptom severity (child and mother reports), child self-reported OCD symptom severity, parental accommodation (child and mother reports), and mother’s self-rated anxiety/depression symptoms. A one-tailed test was selected, not a two-tailed test, because the hypothesized effect direction was expected to be symptom reduction rather than exacerbation. Therefore, a one-tailed test provided greater statistical power and was appropriate given the clear hypothesized direction and methodological standards (55). Significance values were adjusted for multiple comparisons using the False Discovery Rate (FDR) adjustment (56). Change scores deviating more than 3 Median Absolute Deviations (MAD) from the median were excluded as outliers^2^ (57).

Lastly, we explored potential associations between baseline sociodemographic factors and pre-to-post symptom reductions. This analysis was performed using a series of stepwise regressions adjusted for the False Discovery Rate within the model, where the dependent variable was the pre-to-post change in measures showing significant symptom reduction. Models included pre-treatment baseline scores to evaluate the effect of initial symptom severity and control for potential regression to the mean. As we conducted nine separate models, adjusted for FDR within the model, we consider p-values smaller than 0.0055 as significant. We included in our analyses only treatment completers who provided full or partial data, pre and post, and confirmed that there were no differences in baseline measures between completers and non-completers. We did not use data imputation techniques because we had only two data points, and missing one made it impossible to accurately predict the individual’s trend, rendering imputation ineffective.

Analyses were conducted using the ‘lmerTest’, ‘psych’, and ‘stats’ packages in R, and depicted with ‘ggplot2’ and ‘ggsignif’ (58–62).

## Results

### Treatment acceptability and feasibility

**Figure 1:**
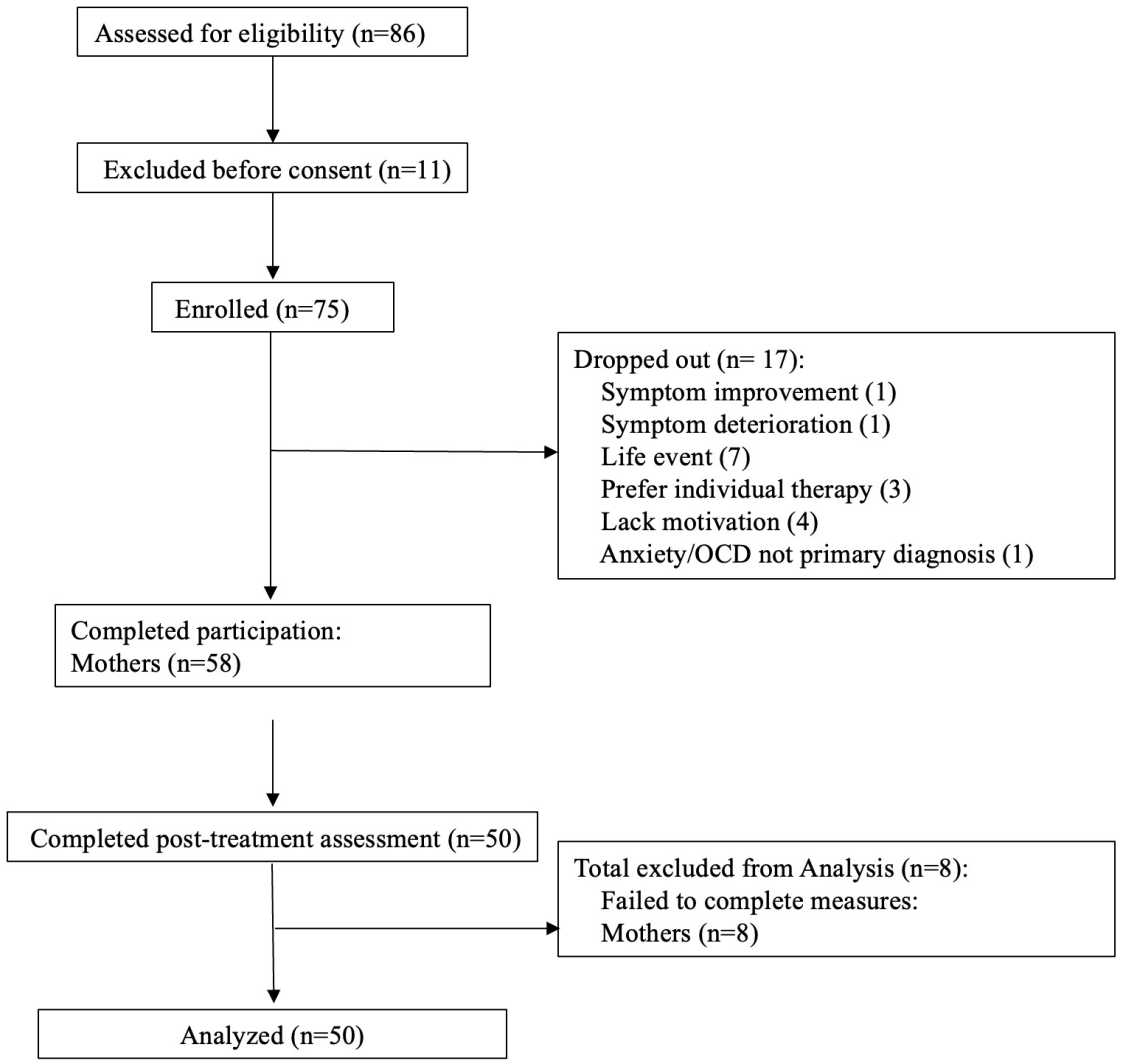
Participants flow through the SPACE group treatment: numbers referred, declined, enrolled, dropped out, and completed treatment. Fathers (n=27) were excluded from the flow diagram due to low completion rates of measures.

Eighty-six parents of children diagnosed with an anxiety disorder and/or OCD were referred to the online SPACE group treatment. Eleven (12.8%) declined SPACE treatment, and 75 parents enrolled. Parents of seventeen children (19.7%) dropped out within the first three sessions (see **Figure 1** for participant flow and treatment dropout reasons), and parents of 58 (77.3%) completed treatment. Eight participants completed treatment without completing questionnaires (both pre and post-treatment) and were excluded from analyses. Fifty mothers and children who completed treatment (66.7% of participants who began therapy), provided full or partial data. Partial child self-report data were primarily due to child refusal to complete pre- or post-treatment questionnaires, or due to parental anxiety interfering with data collection. In such cases, group therapists provided psychoeducation consistent with the SPACE treatment framework, emphasizing parental self-regulation and reducing accommodation of the child’s avoidant behavior. Since only 6 fathers completed self-report measures, father reports were excluded from the current analysis. Demographic and clinical characteristics did not differ significantly between those who completed treatment and those who dropped out. Among treatment completers, no significant age or sex differences were detected between participants who completed post-treatment questionnaires and those who did not (clinical measures could not be tested as these participants did not fill out pre-treatment questionnaires).

Children in the sample (N=50) were 11.2 ± 3.1 years old; 26 females (52.0%) and 24 males (48.0%). Parents’ attendance in the group was high, with an attendance rate of 82.9% (SD=11.5) in group sessions. Almost all (96.2%) of parents reported satisfaction with the telemedicine method of treatment delivery (55% strongly agreed and 41.2% agreed).

### Pre to post child anxiety symptoms

**Table 3** summarizes the pre- to post-treatment changes in child anxiety symptoms as reported by both mothers and children. Mother reports showed the largest symptom reduction for children’s generalized anxiety (t_(45)_=4.42, p_adj_=.001, d [95% CI]=0.65 [0.33,0.97]), followed by separation anxiety (t_(45)=_3.97, p_adj_=.001, d=0.59 [0.27,0.90]), social anxiety (t_(45)=_3.36, p_adj_=.005, d=0.50 [0.19,0.80]), overall anxiety score (t_(45)_=3.22, p_adj_=.001, d=0.47 [0.17,0.78]), school anxiety (t_(45)_=2.47, p_adj_=.017, d=0.36 [0.06,0.66]), and panic/somatic symptoms (t_(45)_=1.96, p_adj_=.049, d=0.29 [-0.01,0.58]). Children’s self-reports showed significant reduction in separation anxiety (t_(35)_=2.82, p_adj_=.010, d=0.47 [0.12,0.81]) and overall anxiety score (t_(35)_=1.95, p_adj_=.049, d=0.32 [-0.01,0.66]), and marginally significant reduction in panic/somatic symptoms (t_(34)_=1.88, p_adj_=.052, d=0.32 [-0.02,0.66]).

**Table 3:** Pre to post outcomes.

Measure	Report	Pre	Post	t	df	p	p_adjusted_	d (95% CI)
**SCARED – Total score**	Mother	31.15	22.72	3.22	45	0.001	0.005	0.47 (0.17, 0.78)
SCARED – Social anxiety	Mother	6.76	5.54	3.36	45	0.001	0.005	0.50 (0.19, 0.80)
SCARED – Separation anxiety	Mother	7.61	5.67	3.97	45	0.000	0.001	0.59 (0.27, 0.90)
SCARED – School anxiety	Mother	1.70	1.11	2.47	45	0.009	0.017	0.36 (0.06, 0.66)
SCARED – Panic/somatic	Mother	5.93	4.20	1.96	45	0.028	0.049	0.29 (-0.01, 0.58)
SCARED - Generalized anxiety	Mother	8.15	6.15	4.42	45	0.000	0.001	0.65 (0.33, 0.97)
**SCARED – Total score**	Child	30.11	25.97	1.95	35	0.030	0.049	0.32 (-0.01, 0.66)
SCARED – Social anxiety	Child	6.64	6.25	0.59	35	0.279	0.279	0.10 (-0.23, 0.43)
SCARED – Separation anxiety	Child	7.64	5.94	2.82	35	0.004	0.010	0.47 (0.12, 0.81)
SCARED – School anxiety	Child	2.03	1.89	0.70	35	0.246	0.264	0.12 (-0.21, 0.44)
SCARED – Panic/somatic	Child	6.47	5.53	1.88	34	0.034	0.052	0.32 (-0.02, 0.66)
SCARED - Generalized anxiety	Child	7.67	6.58	1.50	35	0.072	0.095	0.25 (-0.08, 0.58)
**FASA**	Mother	12.09	8.87	3.04	44	0.002	0.006	0.45 (0.14, 0.76)
FASA (on mother)	Child	11.33	10.08	1.48	35	0.074	0.095	0.25 (-0.09, 0.58)
FASA (on father)	Child	6.87	7.05	0.68	36	0.249	0.264	0.11 (-0.21, 0.44)
**OCI**	Child	12.84	11.28	1.33	24	0.098	0.118	0.27 (-0.14, 0.66)
**BDI**	Mother (on self)	8.48	6.45	2.68	39	0.005	0.012	0.42 (0.10, 0.74)
**BAI**	Mother (on self)	11.06	7.81	3.07	45	0.002	0.006	0.45 (0.15, 0.75)

Means and SD refer to individuals who completed both time points. Degrees of freedom (df) vary due to differential questionnaire completion by parents and children. Sample sizes vary across measures due to differential completion. Ns for child self-report measures were: SCARED subscales (N = 35), except Panic/Somatic subscale (N = 34); FASA-Mother (N = 35); FASA-Father (N = 36); OCI (N = 24); BDI (N = 39); BAI (N = 45). Each analysis uses maximum available data. SCARED, the Screen for Childhood Anxiety Related Emotional Disorders; FASA, Family Accommodation Scale-Anxiety; OCI, Obsessive-Compulsive Inventory; BAI, Beck Anxiety Inventory; BDI, Beck Depression Inventory; 95% CI, 95% Confidence Interval.

Bolded text reflects abbreviations of questionnaire names for total scores only.

### Pre to post maternal symptoms (BDI, BAI, FASA-PR)

Mothers reported reduction in their own anxiety (t_(45)_=3.07, p_adj_=.006, d=0.45 [0.15,0.75]) and depression (t_(39)_=2.68, p_adj_=.012, d=0.42 [0.10,0.74]). There was a reduction in accommodation behaviors, based on mother-reports (t_(44)_=3.04, p_adj_=.006, d=0.45 [0.14,0.76]). Child-reported accommodation reduction was not significant.

### Associations between symptom severity, sociodemographic factors and symptom reduction

Nine separate stepwise regression models predicted symptom reduction (delta scores) for outcomes that significantly improved from pre- to post-intervention^3^. Independent variables included socioeconomic status, specific diagnosis, total number of diagnoses, baseline (pre-treatment) scores, child age and gender, and parents’ occupational and marital status (**Table 4**).

**Table 4:** Stepwise regressions to examine the relationship between T1 symptom severity, sociodemographic factors and symptom reduction (the outcome variable).

Independent variable	B	Std. Error	t value	p	Adjusted p
Model 1 – outcome: SCARED GAD (mother report)					
Social anxiety diagnosis		0.71	0.37	1.90	.06
Overall diagnoses		-0.28	0.14	-1.92	.06
SES		0.19	0.09	2.14	.038
Model 2 – outcome: SCARED separation anxiety (mother report)					
T1 separation anxiety (scaled)		-0.42	0.13	-3.24	.00
OCD diagnosis		-0.66	0.34	-1.95	.06
Overall diagnoses		-0.20	0.13	-1.51	.14
SES		0.16	0.08	1.99	.053
Model 3 – outcome: SCARED social anxiety (mother report)					
Separation anxiety diagnosis	-0.53	0.38	-1.41	.17	.167
Specific phobia diagnosis	-2.71	0.93	-2.91	.006	.023
SES	0.15	0.09	1.77	.08	.17
Model 4 – outcome: total SCARED score (mother report)					
T1 total SCARED score (scaled)	-0.46	0.11	-4.00	<.001	.002
Gender: male (child)	-0.45	0.20	-2.19	.035	.09
Specific phobia diagnosis	-1.49	0.76	-1.95	.06	.09
OCD diagnosis	-0.53	0.26	-2.01	.052	.09
SES	0.11	0.07	1.59	.12	.16
Both parents unemployed	1.01	0.68	1.48	.15	.17
One parent employed	-0.84	0.41	-2.05	.047	.09
Model 5 – outcome: BAI (mother anxiety self-report)					
T1 BAI (scaled)	-0.58	0.12	-4.88	<.001	<.001
Gender: male (child)	0.33	0.24	1.40	.17	.25
Model 6 – outcome: FASA accommodation (mother report)					
T1 FASA score (scaled)	-0.49	0.13	-3.84	<.001	.001
Gender: male (child)	0.39	0.26	1.52	.14	.20
Model 7 – outcome: SCARED separation anxiety (child report)					
Separation anxiety diagnosis	-1.05	0.42	-2.51	.017	.034
Model 8 – outcome: BDI (mother depression self-report)					
T1 BDI score (scaled)	-0.39	0.15	-2.64	.012	.036
Specific phobia diagnosis	-1.02	0.66	-1.54	.33	.20
Model 9 – outcome: SCARED school anxiety (mother report)					
T1 school anxiety (scaled)	-0.50	0.13	-4.00	<.001	.001
OCD diagnosis	-0.61	0.33	-1.86	.07	.11

SCARED, the Screen for Childhood Anxiety Related Emotional Disorders; FASA, Family Accommodation Scale-Anxiety; OCI, Obsessive-Compulsive Inventory; BAI, Beck Anxiety Inventory; BDI, Beck Depression Inventory.

There were positive and significant associations between symptom severity at baseline and pre-to-post symptom reduction, for FASA (β=-0.49, p_adjusted_=.001), SCARED total score (β=-0.46, p_adjusted_=.002), school anxiety (β=0.50, p_adjusted_<.001), and Mother BAI (β=0.58, p_adjusted_<.001). Separation Anxiety diagnosis predicted greater reduction of separation anxiety symptoms, according to child report (β=-1.05, p_adjusted_=.034), while diagnosis of Specific Phobia at baseline predicted reduction in social anxiety symptoms, according to mother report (β=-2.71, p_adjusted_=.023). However, as significance values for these associations were greater than the adjusted.05 cutoff, it is possible that these associations resulted from a type-1 error.

## Discussion

This study suggests overall acceptability and feasibility, as evidenced by a 77.3% treatment completion rate, which is consistent with the 77% completion rate reported in a previous study of in-person group-based SPACE treatment (30). The attrition rate was lower than typically reported for child mental health services in public health settings (63). Nevertheless, individual parent “booster” sessions were deemed clinically necessary on some occasions (i.e., 1–3 sessions on average per group), underscoring challenges in meeting the individual needs of all participants. Findings were associated with significant reductions in maternal anxiety, depressive symptoms, and parental accommodation. Mother reports showed substantial reductions in child overall anxiety symptoms and across specific domains of generalized anxiety, separation anxiety, social anxiety, and school anxiety, while child self-reports showed reductions in separation anxiety. Effect sizes for accommodation, parental distress, and child symptom severity (Cohen’s d ≈ 0.52–1.30) suggest moderate-to-large impacts, aligning with meta-analytic findings from parent-focused telehealth interventions (d ≈ 0.52–0.68) (64). However, significant change was not detected in child-reported maternal accommodation. This finding aligns with previous research demonstrating low parent–child agreement in reports of family accommodation, which has been attributed to divergent perceptions, motivations, and thresholds for identifying accommodating behaviors (65). More broadly, discrepancies between parent and child reports of mental health symptoms are well-documented. Such differences are often shaped by developmental factors, emotional insight, and informant context. For instance, discrepancies between parent and child reports have been found to reflect the child’s perception of relationship quality and closeness, with greater perceived communication and parental acceptance associated with higher agreement in symptom ratings (66). A meta-analysis by Duhig et al. (67) found particularly low agreement between parent and child reports for internalizing symptoms, which are often less observable and more dependent on the child’s emotional awareness and willingness to disclose. These discrepancies have been associated with factors such as child age, insight, communication patterns, and parental sensitivity. This highlights the value of using multiple informants to capture diverse perspectives. In the context of SPACE, it underscores the importance of integrating both parent and child feedback to assess change mechanisms more comprehensively.

The current study extends prior adaptations of SPACE treatment to group settings (30) by delivering it via remote video teleconference. High participant satisfaction with the group setting and telemedicine format aligns with previous literature (68), as reflected in quantitative satisfaction ratings as well as open feedback e.g., “It was effective, the group … Zoom was an excellent solution.” Social support is crucial in parenting, providing emotional and instrumental support and mediating stress effects (69). Parent group interventions enhance peer modeling, bonding, encouragement, normalization, and validation (70–73). These benefits were evident in participant statements, including, “You hear the problems that everyone has, and you say, ‘Wow, maybe my child isn’t crazy’”. This parent’s statement highlights the benefit of normalizing child anxiety-related reactions, which likely contributed to reduced parental accommodation and, consequently, to reduced child symptoms (74). Positive modeling was also evident, as noted by one participant: “I said, everyone’s jumping into the water, we’ll jump into the water too.” These quotes illustrate how group-based treatment benefits were preserved in the telemedicine format. While delivering CBT remotely to children has posed certain challenges (75), our findings align with prior research (76), suggesting that parents may adapt more easily to teletherapy.

Despite high satisfaction, some participants wanted more personalized attention, as one stated, “I have some kind of difficulty … the meeting itself is also ‘Zoomed in’, and the meeting is also a group one, and there is something that makes it difficult to go into the details and the more individual things that are linked to each one.” This preference aligns with previous studies showing some patients’ preference for individual over group-based treatment (77). The rapid transition to telehealth introduced new challenges, including ensuring confidentiality, interpreting nonverbal communications, and dealing with technological issues such as stable internet access and maintaining a distraction-free environment (38, 78, 79). To address these, individualized feedback and “booster” sessions were provided as needed, although, as previously mentioned, most participants did not require such boosters.

SPACE delivered via telemedicine may offer an effective alternative for children who may have difficulty benefiting from remote CBT, including those with attention and memory issues (80). In this study, most children had comorbid cognitive, communicative, or learning disorders (56%: 44% ADHD; 8% Learning Disorders; 4% Speech/Language disorders), supporting the benefit of parent-based remote treatment for these groups. The high comorbidity observed in this sample aligns with findings from a meta-analysis indicating that, in high-income countries, 26.5% of children with mental health disorders are diagnosed with multiple conditions (81). This underscores the need for evidence-based treatments that effectively address the high comorbidity typically met in public health settings.

Results also point to meaningful improvements in mother’s mental health, as indicated in reduced maternal anxiety and depression scores. Parents’ ability to regulate their own stress plays a critical role in shaping children’s coping mechanisms and overall mental health during stressful experiences (82). The reduction in maternal anxiety and depressive symptoms, as well as accommodation, was associated with a decrease in child anxiety symptoms (83, 84). Likewise, it has been suggested that improvement in child anxiety symptoms may be associated with change in parental variables. Therefore, further research on the directionality (85) of symptom change in parent-child anxiety treatment is needed to deepen our understanding of the mechanisms driving these changes.

Mother reports indicated significant improvements in child anxiety symptoms on most measures, while child self-reports showed significant reductions specifically in separation anxiety. This finding aligns with prior research suggesting that separation anxiety is particularly sensitive to parental accommodation (86), as it directly impacts parent-child interactions (86). It makes sense that mothers, being most affected by and attuned to separation anxiety, would report notable changes in this domain. However, the absence of broader self-reported improvements by children immediately post-treatment does not preclude future benefits. Studies have shown that the effects of parent-focused interventions, such as those targeting reductions in parental accommodation, often extend beyond the active phase of treatment as parents implement changes over time (87). This raises the possibility that children may experience and report greater symptom reduction in additional domains as these parental changes become more consistent and ingrained. Clinically, this pattern reinforces the importance of follow-up assessments and potentially extending support to help children internalize and consolidate changes initiated by parental shifts.

Several limitations must be considered. This study employed a retrospective design, analyzing data gathered during standard clinical practice. As such, an *a priori* power analysis was not conducted, and inclusion criteria reflected real-world service conditions. To mitigate concerns of type I errors, we adjusted our results for multiple comparisons. Given this, the modest sample size of 50 mothers may limit the generalizability and statistical power of the findings, especially in the context of multiple comparisons and in light of our alpha corrections. The lack of a control group precludes drawing robust causal conclusions about the intervention’s effectiveness. Future studies using randomized or controlled comparisons (e.g., waitlist or in-person SPACE) are essential to clarify the specific contribution of the telehealth format. The absence of fathers’ self-report data represents a notable limitation, as parental accommodation is common across both parents (88), and paternal accommodation has been shown to predict poorer treatment outcomes independently of maternal accommodation (89). Inclusion of both parents is essential for a more comprehensive understanding of family dynamics, such as influencing child anxiety and treatment outcomes (23). Reliance solely on maternal reports may obscure distinct patterns of accommodation and disproportionately reflect maternal experiences and responses (46). Moreover, emerging evidence suggests that compared to mothers, who tend to exhibit overprotective behaviors, Fathers exert a distinct influence on the development and maintenance of child anxiety through their behaviors, expectations, and often more demanding parenting styles (90). Omitting paternal data may therefore bias results toward maternal patterns, limiting insight into the full range of parental influences on child anxiety. Future strategies should incorporate evidence-based approaches to actively engage fathers—such as direct invitations, father-specific content, and flexible delivery formats including evening scheduling, telehealth, or shorter, targeted sessions (91)—to reduce barriers to participation and acknowledge fathers’ unique contributions to family functioning and treatment outcomes (92). The sample also consisted exclusively of Hebrew-speaking Israeli mothers from a single public hospital, which may limit the generalizability of findings to other cultural, linguistic, or socioeconomic contexts. In addition, we did not assess the specific characteristics of patients who required booster sessions. As a result, we are limited in our ability to make targeted recommendations regarding the implementation and optimal use of booster sessions based on individual patient needs. Finally, the lack of follow-up data prevents assessment of long-term effects.

Despite these limitations, this study provides valuable insights into the novel adaptation of an evidence-based parent group treatment within the public health sector. The heterogeneity of the sample, with a broad age range and highly comorbid patient profiles, enhances the generalizability of the findings to diverse populations and supports the applicability of such treatments for children and adolescents with anxiety disorders and/or OCD under realistic conditions. These findings suggest that SPACE, adapted for telehealth, could effectively treat a wide range of patients in the public health sector, contributing to reducing barriers to effective evidence-based interventions.

## Data Availability

The raw data supporting the conclusions of this article will be made available by the authors, without undue reservation.

## Footnotes

1 Variables that deviated from normality had more than 30 observations, which, according to the central limit theorem, suggests that the sampling distribution of the mean approximates normality and justifies the use of parametric tests. The only variable with fewer than 30 observations (OCI child) was confirmed to be normally distributed based on the Shapiro-Wilk test.

2 Three observations were excluded as outliers: one from the mother’s BAI, one from the child’s father’s FASA, and one from the child’s SCARED panic/somatic subscale. A sensitivity analysis including these outliers yielded a nonsignificant reduction in BAI (adjusted p=.08).

3 Mother report on child: SCARED GAD, SCARED separation anxiety, SCARED social anxiety, SCARED total score, FASA; Child self-report: SCARED separation anxiety; Mother self-report: BAI, BDI.
